# Early Taste Experiences and Later Food Choices

**DOI:** 10.3390/nu9020107

**Published:** 2017-02-04

**Authors:** Valentina De Cosmi, Silvia Scaglioni, Carlo Agostoni

**Affiliations:** 1 Valentina De Cosmi Pediatric Intensive Care Unit, Fondazione IRCCS Cà Granda Ospedale Maggiore Policlinico, Branch of Medical Statistics, Biometry, and Epidemiology “G. A. Maccacaro”, Department of Clinical Sciences and Community Health, University of Milan, 20122 Milan, Italy; valentina.decosmi@gmail.com; 2 Silvia Scaglioni Fondazione De Marchi Department of Pediatrics, Fondazione IRCCS Cà Granda Ospedale Maggiore Policlinico, 20122 Milan, Italy; silviascaglioni50@gmail.com; 3 Carlo Agostoni Pediatric Intermediate Care Unit, Fondazione IRCCS Cà Granda Ospedale Maggiore Policlinico, Department of Clinical Sciences and Community Health, University of Milan, 20122 Milan, Italy

**Keywords:** early taste, food preferences, breastfeeding, complementary feeding, feeding strategy, children obesity, food choices

## Abstract

Background. Nutrition in early life is increasingly considered to be an important factor influencing later health. Food preferences are formed in infancy, are tracked into childhood and beyond, and complementary feeding practices are crucial to prevent obesity later in life. Methods. Through a literature search strategy, we have investigated the role of breastfeeding, of complementary feeding, and the parental and sociocultural factors which contribute to set food preferences early in life. Results. Children are predisposed to prefer high-energy, -sugar, and -salt foods, and in pre-school age to reject new foods (food neophobia). While genetically determined individual differences exist, repeated offering of foods can modify innate preferences. Conclusions. Starting in the prenatal period, a varied exposure through amniotic fluid and repeated experiences with novel flavors during breastfeeding and complementary feeding increase children’s willingness to try new foods within a positive social environment.

## 1. Introduction

Childhood is a period of very rapid growth and development. In this critical phase, food preferences are formed, are tracked into childhood and beyond, and foundations are laid for a healthy adult life [1]. The characterization of feeding practices is important for the determination of which factors of the early environment can be modified and thus are amenable to intervention. Since early life exposures may contribute to the risk of obesity [2], the topic is highly recognized to be of social and public health interest [3,4].

**Figure 1:**
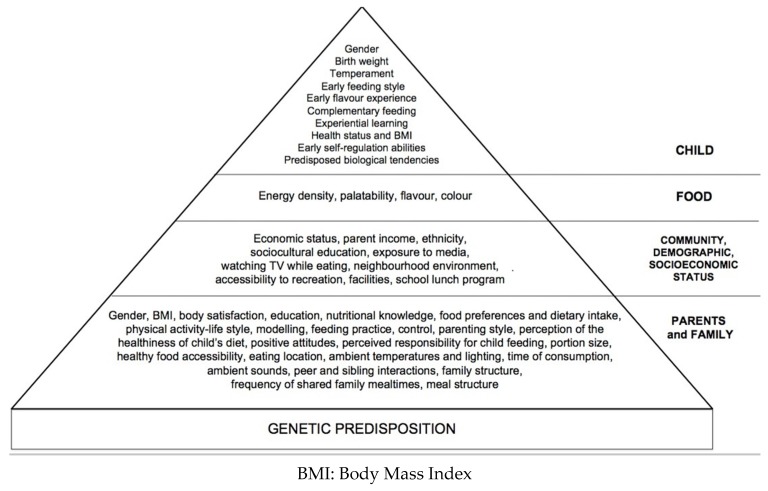
Environmental factors that influence child eating behavior.

Infants’ and children’s eating and activity behaviors are influenced by both intrinsic (genetics, age, gender) and environmental (family, peers, community, and society) factors [5]. These factors are fully displayed in Figure 1.

Firstly, prenatal exposure, and then breastfeeding, have been associated with flavor stimulation and moderately lower childhood obesity risk in many studies [2,6,7]. Later on, the period of complementary feeding is also crucial, both for obesity prevention and for setting taste preferences and infant attitude towards food. Parents act by teaching children in different ways how, what, when, and how much to eat and by transmitting cultural and familial beliefs and practices surrounding food and eating [8]. Parents’ influence is significant: it is reflected both by what is on the plate and the context in which it is offered [9].

Obesity is a burden social disease, linked to lifestyle and food choices changes, characterized by low level of physical activity, high energy density, and free sugar-rich food. As nutritional habits are tracked from infancy to adulthood, we investigated factors inside the child milieu, possibly connected to flavor learning and feeding practices. In particular, we focused on strictly child-related factors. Parental influence is only described in terms of food offering feeding style, while parental modeling is not a topic of our review. We reviewed (1) the biological and social early-life exposures; (2) the prenatal influence of the amniotic fluid; (3) how breast milk and formula may influence taste development; (4) the role of complementary feeding; (5) the parental and sociocultural factors associated with trajectories of health in adulthood.

## 2. Methods—Literature Search Strategy

Electronic databases (Pubmed, Medline, Embase, Google Scholar) were searched to locate and appraise relevant studies. We carried out the search to identify articles published in English on the relation between children’s early taste experiences and their food choices during childhood. Relevant articles published after 2005 and up to August 2016 were identified using the following search words in various combinations. The literature search was not aimed to conduct a systematic review or meta-analysis of all of the available literature on this topic, but to explore the pertinent observations in a period of 10 years. Our work is a narrative review, and search terms were inserted individually and using the booleans AND and OR. The following terms were included in the search strategy: (“early taste experiences” OR “early food preferences”) & (“food choices in childhood”) OR (“parental feeding practices” OR “parent’s feeding strategies” OR “parental modeling”) & (“family environmental factors” OR “family eating environments”) & (“early exposure” AND “obesity risk” AND “childhood obesity risk factors”) & (“amniotic fluid” OR “breast milk” AND “taste AND flavor development”) & (“early diet experiences” OR “development of eating habits”) & (“Food choices”). More than 5000 references matched the terms of the search, and around 1500 had been published in the past 10 years. The authors selected the articles and assessed the potentially relevant ones.

### 2.1. Effects of Early Taste Experiences

According to a working hypothesis, the first thousand days of life represent a sensitive period for the development of healthy eating habits, and for this reason, interventions are likely to have a strong impact on health outcomes later during childhood and adulthood. This critical period starts with feeding through the cord during gestation, passes toward oral feeding with milk, and then the complementary feeding begins and the infant discovers a variety of foods and flavors. Humans generally have inborn positive responses to sugar and salt, and negative responses to bitter taste [10]. Genetically determined individual differences also exist, and interact with experience to ensure that children are not genetically restricted to a narrow range of foodstuffs [11]. Children are also predisposed to prefer high-energy foods, to reject new foods, and to learn associations between food flavors and the post-ingestive consequences of eating [12]. This genetic predisposition appears to have evolved over thousands of years when foods—especially those high in energy density—were scarce. Few children—PROP (6-*n*-propylthiouracil) tasters—are sensitive to bitter taste and have higher liking and consumption of bitter foods, such as cruciferous vegetables. Additionally, those children who are unable to taste PROP (nontasters) like and consume more dietary fat and are prone to obesity; thus, genetic variation in the ability to taste bitter compounds may have important implications as a marker for dietary patterns and chronic health in children. The available literature suggests that some children may require additional strategies to accept and consume bitter-tasting fruits and vegetables and that genetic predisposition may be modified by repeated exposures [13,14].

### 2.2. Amniotic Fluid and Breast Milk

The ability to recognize a variety of flavors involves multiple chemosensory sensations, primarily the sense of taste and smell. Food experiences begin prenatally, since chemosensory systems have an adaptive and evolutionary role and are functional before birth [10]. The exposure to an in utero environment may cause permanent effects on the developing tissue. These effects are referred to as “programming”, and are important risk factors for chronic diseases in later adulthood [15].

Children usually prefer foods that are high in sugar and salt over those which are sour and bitter tasting, such as some vegetables. Preferences for salt and the refusal of bitter can be modified early through repeated exposure to flavors in amniotic fluid, mother’s milk, and solid foods during complementary feeding. Flavor senses are well developed at birth, and continue to change throughout childhood and adolescence, serving as gatekeepers throughout the life span, controlling whether to accept or reject a foreign substance. Since amniotic fluid and breast milk both reflect to a variable degree the food composition of the maternal diet, a repeated exposure to their flavors increases infants’ acceptance of foods [16]. While the knowledge of the influence of the maternal diet on breast milk is mostly indirect [17], the sensory experiences with food flavors in mothers who ate a varied diet may explain why their breastfed children tend to be less picky [18] and more willing to try new foods during childhood [11,19,20]. A cohort study [21] on 1160 mother–infant pairs showed that preponderance of breastfeeding in the first 6 months of life and breastfeeding duration were associated with less maternal restrictive behavior and less pressure to eat. Accordingly, compared with bottle-feeding, breastfeeding may promote maternal feeding styles that are less controlling and more responsive to infant cues of hunger and satiety, thereby allowing infants to develop a greater self-regulation of energy intake [21].

### 2.3. Formula-Fed Infants

The early flavor experience of formula-fed infants is markedly different from that of breast-fed infants. Exclusively formula-fed children do not benefit from the ever-changing flavor profile of breast milk. Their flavor experience is more monotone and lacks the flavors of the foods of the mother’s diet. There are striking differences in flavors among the different types of formulas and brands of formulas, and formula-fed infants learn to prefer the flavors of the formula they are fed and foods containing these flavors [11]. There is a plethora of infant formulas on the market that differ in macronutrient composition. When evaluating the effect of diet composition on growth and health outcomes, it may no longer be appropriate to consider all formula-fed infants as a homogeneous group, because infant formulas may also differ in both fat and carbohydrate composition/structure as well as protein composition, and these differences may in turn affect growth and flavor development [22]. Consequently, it is important to understand the composition of the diet to which breastfeeding is being compared before drawing conclusions. European and US populations reveal an association between breastfeeding and a reduced prevalence of obesity in a meta-analysis; however, in a large randomized controlled trial, there was no effect of breastfeeding on body mass index in later childhood [23]. When infants are fed with a formula that is more similar in protein content to breast milk (lower vs. higher protein), their weight-for-length at 24 months of age does not differ from breastfed infants [24]. Another difference is found in infants consuming protein hydrolysate formula when compared with cow’s milk formula: they are satiated sooner and have a less excessive rates of weight gain [25]. The mechanism of this effect is currently unknown, but is hypothesized to be related to differences in free glutamate (which is abundant in human breast milk) [26,27].

### 2.4. Complementary Feeding and Future Consumption of Fruits and Vegetables during Childhood

Early learning about flavours continues during the complementary feeding period, through the introduction of solids and changing exposures to a variety of new foods. In this peculiar time of the child’s life, there is the transition from breast/formula feeding to a complementary solid diet, and infants discover the sensory (texture, taste, and flavour) and nutritional properties (energy density) of the foods that will ultimately compose their adult diet [28]. Being exposed to a variety of foods during the complementary feeding period helps modulate the acceptance of new foods in the first year, whereas exposure in the second year may have a more limited impact [29].

Young children (especially 2–5 years old) exhibit heightened levels of food neophobia during this time. This means that they are unwilling to eat novel foods; it is interpreted as an adaptive behaviour, ensuring children consume foods that are familiar and safe [30].

Distaste—dislike of the sensory characteristics of a food—appears to be the strongest driver of neophobia in young children [31]. Indeed, the two strongest predictors of young children’s food preferences are familiarity and sweetness, reflecting unlearned preferences. However, these innate tendencies are paired with a predisposition to learn from early experiences through associative learning and repeated exposure, allowing the child to learn how to accept and prefer the foods that are available within his particular environment [30]. Repeated exposures to a food increase their familiarity, and it is one of the primary determinants of its acceptance. Several studies have shown that a food is consumed more and is judged as more liked by the infant after several offers. For instance, an increase in acceptance of a new green vegetable was observed after at least eight exposures to this food [31]. The effect of repeated exposure is potent enough to increase the acceptance of foods which had been previously identified by the mother as being refused by her infant during the beginning of the complementary feeding, which were most often green vegetables, but also pumpkin [32]. However, despite the efficacy of this mechanism, foods are most often only presented a limited number of times (often less than five times) before the parents decide that the infant dislikes this food [33,34,35].

Reactions towards new foods differ according to food groups [28]. Lange et al. (2013) asked mothers to report their infant’s reactions to new foods at the beginning of complementary feeding, and they found that fruits and vegetables, which are firstly offered to infants, are less accepted than other food groups [36].

A study of de Launzon et al. investigated the long-term effects of early parental feeding practices on fruit and vegetable intake. The study used data from four European cohorts, in which data on fruits and vegetables consumption were assessed with a questionnaire. These cohorts reported different findings. Fruit and vegetable intake in early childhood varied with an average intake of <1 vegetable/day in the Greek EuroPrevall study and >3 vegetables/day in the Generation XXI Birth Cohort. Moreover, longer breastfeeding duration was found in Generation XXI than in the others. The timing of complementary feeding varied too: complementary foods were introduced mainly between 3 and 4 months of age in ALSPAC (British Avon Longitudinal Study of Parents and Children), at ≈4 months in Generation XXI, and at ≈5 months in Greek Euro-Prevall. In EDEN (French Etude des De’terminants pre et postnatals de la sante’ et du de’veloppement de l’Enfant), there was no peak age for introduction to complementary foods.

A concordant positive association between breastfeeding duration and fruit and vegetable intake was found in different cultural contexts, with a longer breastfeeding duration consistently related to higher fruit and vegetable intake in young children, whereas the associations with age of introduction to fruit and vegetable intake were weaker and less consistent [37].

Similarly, 2- to 8-year old children who were breastfed for three or more months were more likely to eat vegetables, as compared to children who were breastfed for a shorter time [28,38]. Taste may impact the acceptance of new foods, since vegetables added with salt or a salty ingredient are more easily accepted [39]. However, this observation should not encourage parents to use salt or salty ingredients, because sodium is not recommended for infants [2,35]. Furthermore, acceptance of green beans appears more difficult than that of carrot, in part due to the difference in the tastes of the two vegetables, since carrots are sweeter than beans [35].

Therefore, the attraction towards new foods in the absence of imprinting and/or learning seems to depend on their tastes and on the sensory properties of foods. At the same time, some individuals may be more sensitive to taste features. In particular, for the sour, sweet, and umami tastes, the individual sensitivity to taste in water solutions at the age of 6 months was predictive of the positive reaction towards foods bearing these tastes [39].

Nicklaus and coworkers in 2014 studied the effect of repeated exposure and of flavor-flavor learning on toddlers’ (2–4 years) acceptance of a non-familiar vegetable, and concluded that repeated exposure is the simplest choice to increase vegetable intake in the short and long term [29,35]. The NOURISH is a randomized controlled trial which evaluated an intervention commencing in infancy to provide anticipatory guidance to first-time mothers on a “protective” pattern of complementary feeding practices that were hypothesized to reduce childhood obesity risk. In agreement with the results, investing in early advice on training mothers about responsive complementary feeding can improve maternal feeding practices, and suggests that complementary feeding practices promoting the self-regulation of intake and preference for healthy foods may have positive effects on obesity risk up to 5 years of age [15,40].

Early experiences with nutritious foods and flavour variety may maximize the likelihood that children will choose a healthier diet as they grow, because they like the tastes and the variety of the foods it contains. A recent investigation demonstrated that early exposure to a rotation of vegetable flavours first added to milk and then to cereals increased the intake and liking of these vegetables. Infants assigned to the intervention ate more of the target vegetables in the laboratory and at home than those assigned to the control group [12].

During childhood, the strongest predictors of what foods young children eat are (1) whether they like how the foods taste; (2) how long they were breastfed and whether their mothers ate these foods; and (3) whether they had been eating these foods from an early age [20,41]. During early childhood, infants are more likely to accept new foods, and parents should promote a varied diet and the child’s curiosity towards food to reduce neophobia in toddlers [41,42]. After the age of 3–4 years, reported dietary patterns/food habits remained quite stable, further highlighting the importance of getting children on the right track from the initial stages of learning to eat [43].

### 2.5. Sociocultural and Family Environment

Social support plays a key role starting from birth. Accordingly, the initiation and continuation of breastfeeding and cultural beliefs—shared through kin, friend, and neighbors networks—may serve to promote or limit breastfeeding [2]. Parents create food environments for children’s early experiences with food and eating, and also influence their children’s eating by modeling their own eating behaviors, taste preferences, and food choices. As children grow and become more independent, familial influences on eating behavior may diminish, and other factors such as those of peers may become more influential [44]. Parents and caregivers play a role in structuring early feeding, which in turn is embedded in the larger micro- and macro-environments that shape parental beliefs, decisions, and practices [45]. It has been shown that forcing a child to eat a particular food will decrease the liking for that food, and that restricting access to particular foods increases rather than decreases preferences [14].

Social influences become increasingly important for the development of food preferences throughout infancy, and may either support or contrast the preferences learned during the prenatal and early postnatal periods [30]. Beauchamp and Moran [46] examined the preference for sweet solutions versus water in approximately 200 infants. At birth, all of the infants preferred sweet solutions to water, but by 6 months of age, the preference for sweetened water was linked to the infants’ dietary experience. Infants who were routinely fed sweetened water by their mothers showed a greater preference for it than did infants who were not. Therefore, offering complementary foods without added sugars and salt may be advisable not only for short-term health but also to set the infant’s threshold for sweet and salty tastes at lower levels later in life [14]. Neophobic tendencies can be reduced and preferences can be increased by exposing infants and young children repeatedly to novel foods. Children need to be exposed to a novel food between 6 and 15 times before increases in intake and preferences are seen. A recent study found that repeatedly exposing children to a novel food within a positive social environment was especially effective in increasing children’s willingness to try it. These findings suggest the importance of both the act of repeatedly exposing children to new foods and the context within which this exposure occurs [30].

## 3. Discussion

The prevalence of childhood obesity is rising, and multiple studies indicate that most of the risk factors develop during the early phases of life. These factors may range from the prenatal to postnatal period.

Within this context, strategies to successfully promote better acceptance of vegetables should be identified. In spite of a huge body of literature, practical aspects and the results of their application are still poorly understood. This is due to the high complexity related to physiological mechanisms underlying early sensory experiences and the development of sensory preferences.

Breast-fed infants more easily accept a new vegetable, and have higher acceptance of new foods as they are introduced into the infant’s diet. There are many factors which influence infants’ feeding behaviours; they interact and contribute to the creation of future eating habits. Mothers who consume an array of healthy foods themselves throughout pregnancy and lactation—and subsequently feed their children these foods at the complementary feeding period—can promote healthful eating habits in their children and families. Although a large part of food-preference development occurs during early childhood, food preferences continue to change during adolescence up to adulthood, and the factors that influence these changes become more complex through the years [30]. While it is emphasized that an excessive intake of foods high in salt and refined sugars early in life may be associated with later non-communicable disorders, the individual genetic background and sensitivity to specific nutrients makes it difficult to substantiate a precise cause and effect dose-dependent relationship.

On the other side, food likes and dislikes are learned, and the learning process begins early and depends on biological and sociocultural attitudes. 

## 4. Conclusions

Attention should be paid to the different socio-cultural contexts of eating in future studies, and cohort studies are needed to quantify the effect of early stimulation of taste and preferences. Randomized controlled trials on early diet, focusing on both caregivers and children’s behaviours and adjusted for food-related genotype are also essential for understanding how preferences can be modified to promote healthful diets across the life course [30].
